# Fishing for Space: Fine-Scale Multi-Sector Maritime Activities Influence Fisher Location Choice

**DOI:** 10.1371/journal.pone.0116335

**Published:** 2015-01-27

**Authors:** Alex N. Tidd, Youen Vermard, Paul Marchal, John Pinnegar, Julia L. Blanchard, E. J. Milner-Gulland

**Affiliations:** 1 SPC, BP D5, 98848, Noumea, New Caledonia; 2 Cefas, Pakefield Road, Lowestoft, Suffolk, NR33 0HT, United Kingdom; 3 IFREMER, Département Ressources Biologiques et Environnement Responsable de l’Unité Halieutique Manche-Mer du Nord, Unit 150, Quai Gambetta, BP 699 62321, Boulogne sur mer, France; 4 Department of Animal and Plant Sciences, University of Sheffield, Alfred Denny Building, Western Bank, Sheffield, S10 2TN, United Kingdom; 5 Department of Life Sciences, Imperial College London, Silwood Park Campus, Ascot, SL5 7PY, United Kingdom; Bangor University, UNITED KINGDOM

## Abstract

The European Union and other states are moving towards Ecosystem Based Fisheries Management to balance food production and security with wider ecosystem concerns. Fishing is only one of several sectors operating within the ocean environment, competing for renewable and non-renewable resources that overlap in a limited space. Other sectors include marine mining, energy generation, recreation, transport and conservation. Trade-offs of these competing sectors are already part of the process but attempts to detail how the seas are being utilised have been primarily based on compilations of data on human activity at large spatial scales. Advances including satellite and shipping automatic tracking enable investigation of factors influencing fishers’ choice of fishing grounds at spatial scales relevant to decision-making, including the presence or avoidance of activities by other sectors. We analyse the determinants of English and Welsh scallop-dredging fleet behaviour, including competing sectors, operating in the eastern English Channel. Results indicate aggregate mining activity, maritime traffic, increased fishing costs, and the English inshore 6 and French 12 nautical mile limits negatively impact fishers’ likelihood of fishing in otherwise suitable areas. Past success, net-benefits and fishing within the 12 NM predispose fishers to use areas. Systematic conservation planning has yet to be widely applied in marine systems, and the dynamics of spatial overlap of fishing with other activities have not been studied at scales relevant to fisher decision-making. This study demonstrates fisher decision-making is indeed affected by the real-time presence of other sectors in an area, and therefore trade-offs which need to be accounted for in marine planning. As marine resource extraction demands intensify, governments will need to take a more proactive approach to resolving these trade-offs, and studies such as this will be required as the evidential foundation for future seascape planning.

## Introduction

As human population growth continues to increase there is a need to balance competing demands for natural resources. Traditionally seen as a common property resource, the sea is confronted increasingly with competition for space by competing sectors, e.g. fisheries, oil and gas exploitation, aggregate extraction, wind energy, shipping and transport, recreation, dumping and the military activities. Spatial planning and the regulation of human activities and pressures at sea are therefore becoming a concern, especially given that some resources are limited in space and quantity. Since 2008, the European Union has placed a responsibility on member states to achieve common principles based on the “Roadmap for spatial planning” [1], which falls under the Integrated Maritime Policy (IMP; [2]), and is generally referred to as Maritime Spatial Planning (MSP). The objectives of MSP are to manage anthropogenic activities in space and time, precluding or minimising conflicts between competing sectors without negatively impacting the ecosystem, operating within the Marine Strategy Framework Directive (MFSD; [3]). However, because sectors at sea can change rapidly and the complexities of natural systems are linked and inter-reliant, a management decision for one sector may affect others, and MSP needs to be treated as a process of continuous, adaptive management process.

Given the importance of MSP, several writers have stressed the importance of fleet-based spatial management in the commercial fisheries sector [4], [5], accounting for different fleet activities at a scale fine enough to be integrated into the MFSD process. To date, integration has been difficult owing to the broad scale (ICES statistical rectangle ∼900 nautical miles^2^) at which some data (e.g. landings) are reported. With the emergence of Vessel Monitoring Systems (VMS) over the past decade, however, MSP is now potentially possible at a finer scale. Issues of data confidentiality between member states have hampered the use of this information, and there is also a historic reluctance of fishers to provide accurate landings information for fear of conceding knowledge of profitable fishing grounds [6], and that the information might be used against their interests by other authorities. For example [7] suggested that fishers are concerned that conservationists might identify productive fishing grounds as suitable for Marine Protected Areas (MPAs), or fisheries managers might implement tighter enforcement constraints. In the light of the limited data availability and confidentiality, fisheries managers are looking now for alternative approaches to assist spatial planning, which will reduce implementation error i.e. where the effects of management differ from that intended [8].

One such approach involves anticipating fisher behaviour in response to regulation. Fisher behaviour cannot be predicted with certainty because of the many factors which influence where and when a fisher will operate. However, if managers can better anticipate fisher behaviour, then they may be able to reduce the unanticipated side-effects of management actions aimed both at the fishery sector and at other sectors. Traditional fisheries management treats fishers as static and homogenous with no consideration of their behaviour and individual aims [9], [10]. Recent studies have applied random utility model (RUM) methodology [11]–[13] to this issue, because such models offer an opportunity to study individual behaviour at a finer scale of space and time than previous approaches [14].

**Figure 1:**
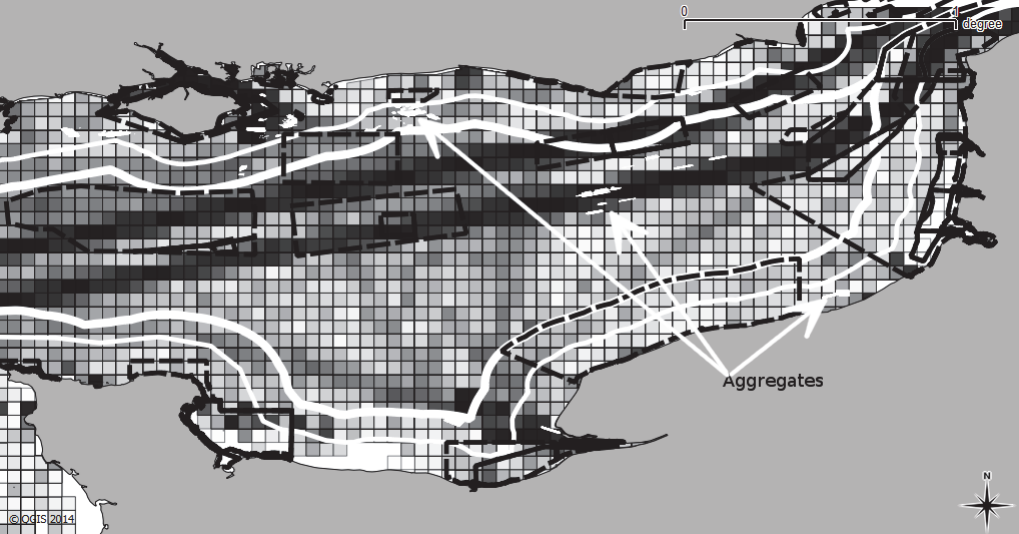
Spatial overlap of sectors (coloured pixels represent maritime traffic densities, thick and thin white lines the12 and 6 mile limits respectively, aggregate mining sites (labelled) and the dashed black line proposed Special Areas of Conservation) within the English Channel.

The objective of the present study was to model the key determinants of where fishers choose to fish, building on retrospective time-series and including interactions with a selection of key sectors also competing for space in the area. In this study we acquired data for the English and Welsh scallop-dredging fleet operating in the eastern English Channel (ICES Division VIId) and these fleets form the basis of our case study. This area also contains one of the busiest shipping lanes in the world, the route between the Atlantic Ocean and the North Sea, which we hypothesise might have a negative impact on commercial fishers. There are also several active marine aggregate extraction sites and fishers have expressed concerns about the accumulation of such sites and the effect of fishing pressure concentrating elsewhere for fear of gear damage, and the sustainability of fish stocks that are already heavily exploited in these areas [15]. The fishing restrictions in the area consist of local English bylaws prohibiting beam trawlers of > = 300 horsepower or 70 gross registered tons from the 12 mile belt of sovereign waters around the English coast to restrict competition with the inshore sole fishing fleet (Fig. 1). This ruling also prevents fishing by any international fishing vessel, though the area can be used for safe passage. There is a further 6 mile restricted zone to assist inshore vessels by prohibiting some fishing vessels of size >14m (depending on which regional Inshore Fishery and Conservation Area—IFCA they fall under) and limitations on scallop vessels with a certain number of dredges. Most of the vessels operating in the region are small (<10 m) inshore boats that deploy gillnets, trawls, longlines, traps and pots, and target sole (*Solea solea*), plaice (*Pleuronectes platessa*), cod (*Gadus morhua*), bass (*Dicentrarchus labrax*) and some skates and rays (Rajidae; [16]).

A mixed logit RUM was developed to analyse the determinants of fisher behaviour at a fine scale using English and Welsh VMS data. This model evaluates the effect of the key potential competing sectors on fishing behaviour. Suggestions are then made as to how the method can be used in integrated MSP in anticipation of the potential establishment of Special Areas of Conservation (SACs) in the area as part of UK commitments to the EU’s Habitats Directive [17] or Marine Conservation Zones (MCZs) under the UK Marine and Coastal Access Act 2009.

## Materials and Methods

### Ethics Statement

I confirm that the author adhered to general guidelines for the ethical use of humans and animals.

### The UK scallop fleet

The UK scallop (*Pecten maximus*) industry is one of the UKs most valuable fisheries and was valued at >£66.9 million (58000 tonnes), £16 million in the Channel alone in 2012 [18], employing >13000 people in the catching sector and 17 000 in the processing sector [19]. Scallops are fished in one of three ways, dredging, trawling and hand-diving. Dredging is the most common method (95% of king scallops landed in UK are caught using dredges [20] and 2–4% of king scallops are hand collected by divers, [21]). Scallop dredges consist of a heavy metal frame with a chain mesh and a set of spring-loaded teeth pointed downwards to assist in raking out the scallops into the dredge’s chain mesh. These dredges are connected to a beam, which in turn is connected to warps that are towed over the seabed by the fishing vessel. Queen scallops (*Aequipecten opercularis*) are typically caught in much the same way however queen scallops are active swimmers and fishers are able to engage in trawling for them during seasonal king scallop dredging restrictions.

Variability in landings and in number of vessels operating, resulting from fluctuations in good recruitment, market demand, regulations and more recently fuel price, are common features of scallop fisheries. Generally current management of scallop fisheries is through minimum landing sizes and the numbers of dredges regulated by local sea fisheries committees, as there are no catch limitations. The UK scallop-dredging fleet is said to be nomadic in nature, moving around the UK coast to fish wherever scallop abundance is best and operating there until those grounds become economically non-viable. They then return a few years later when stocks there have recovered [19]. In recent years, there has been an increase in the number of vessels operating in the eastern English Channel fishery. This may partly be due to more restricted fishing opportunities elsewhere, such as in Cardigan Bay [22], but also the Prohibition of Fishing for Scallops (Scotland) Order 2003 banning the use of more than 14 dredges per side anywhere in Scottish waters [23] hence displacing larger vessels which use a greater number of dredges to other locations. However Defra [19] suggest that this increase is predominantly among the larger (≥15 m long), more powerful, vessels and is also due to an increase in scallop abundance resulting from enhanced recruitment.

### Data

The UK’s Department for the Environment, Food and Rural Affairs (Defra) database for fishing activity and the fleet register were used to select commercial landing and vessel data from the English and Welsh fleet (excluding Scottish and Northern Irish data due to confidentiality issues). Individual trip data for commercial scallopers were collated for the years 2005–2010. The data collected for each vessel included species landed, hours fished, landed weight per ICES statistical rectangle (kg), month of fishing, year of fishing and total value of the catch by species, vessel and trip. Within the EU, it is currently only a requirement for vessels >10 m long to submit logbooks, but the database also contains a subset of catch from <10 m vessels that historically reported their catches.

Methodology for the definition of fleets was based on the European Commission’s Data Collection Regulation (DCR; [24]). VMS monitoring in the European Union [25], [26] has been in place since 2000, initially for fishing vessels of ≥24 m long, post-2005 for vessels ≥15 m long, and in 2012 ≥12 m long. The data are required by regulatory authorities for vessel monitoring purposes (and nowadays, increasingly for scientific research) and are characterised by a ping every 2h giving position, course and speed. Over the past few years, authors such as [27], [28] and [29] have described methods to determine fishing or steaming activities from unprocessed VMS data. No individual method has been adopted as definitive, so the data for the years 2005–2010 were processed as described by [28]. Logbook data and VMS fishing records were combined by vessel and ICES rectangle, forming a detailed dataset of fishing activity. The ICES rectangle was further formatted into 200 (3′ × 3′) squares and all the coordinates from the VMS data were assigned into these spatial units.

Marine diesel prices, excluding value-added tax and duty, were obtained from the UK Department of Energy and Climate Change. Aggregate-extraction intensity data by month for the years 2005–2010 were obtained from the UK’s Royal Haskoning and the Institut Français de Recherche pour l’Exploitation de la Mer. Shipping/transport traffic information was obtained from the Automatic Identification System (AIS) of the UK Maritime Coastguard Agency. UK 6-mile and French 12-mile limits were added to the maritime activities dataset because it was thought that competition for space with the local inshore fleet would be an influencing factor.

### The model

**Figure 2:**
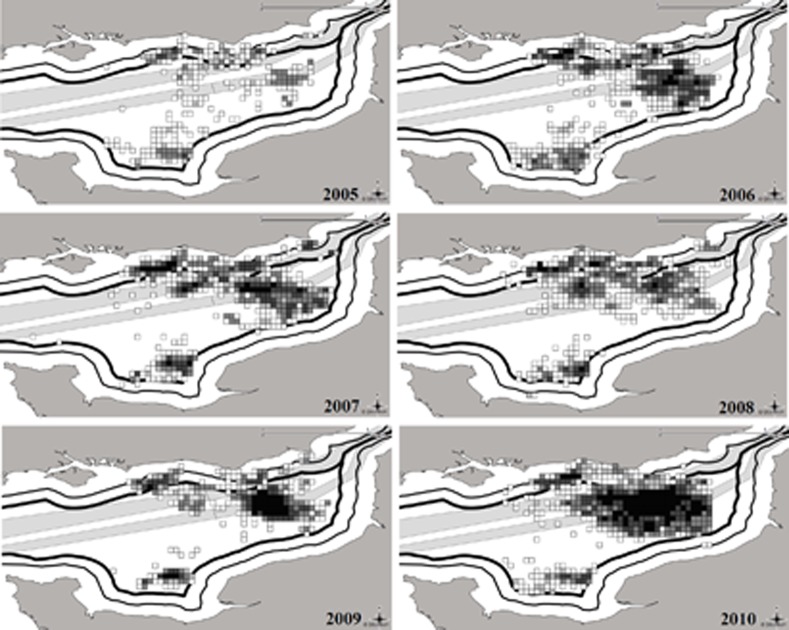
The eastern English Channel displaying total annual scallop dredging effort densities in hours fished (black pixels = 200 hours).

Having populated the dataset with all covariates, we developed a mixed RUM to determine the key determinants of fisher behaviour in relation to competing sectors and fishing-specific covariates. We hypothesise that key competing sectors of activity as well as fishing costs (i.e. fuel price) negatively impact the spatial coverage of fishing operations (as presented in Fig. 2), in contrast to expected vpue and past effort (knowledge or habit) which positively influence fishing operations. Pioneering research by [30], [31] on the use of discrete choice and economics methodologies demonstrated the relationship between utility maximization and discrete choice, where utility influences individual choice with a deterministic and stochastic error component. RUM derives its name from discrete utility maximization and assumes that the choices are random to the analyst. A mixed logit choice RUM was implemented because it relaxes the non-IIA (Independence of Irrelevant Alternatives) assumptions associated with preference heterogeneity among fishers. This approach is efficient in dealing with panel data for repeated individual choices, as is the case within this study. For a detailed explanation of mixed logit, see [32] and [33]. Succinctly, the total utility *μ_njt_* of fisher *n* for site *j* in trip *t* is

$$\mu_{njt}=\beta {'}_{n}x_{njt}+\epsilon_{njt}.$$ (1)

where *β′_n_ x_njt_* represents the observed utility and *ϵ_njt_* is the error distribution that is part-correlated and part independently and identically distributed (iid) over alternatives and individuals[31], [34]. Within the mixed logit framework, *β_n_* was assumed to follow a normal distribution, and for a given value of *n* (for simplicity disregarding *t*), the conditional probability of choice *j* across all other choices *j* = 1 to *J* is estimated by drawing random values *β* by simulation using

$$P_{nj}(\beta_{n})=\frac{\exp(\beta_{n}x_{nj})}{\sum_{j=1}^{J}\exp(\beta_{n}x_{nj})},$$ (2)

where *β_n_* is a vector of coefficients that varies across individuals, and *x_nj_* is a vector of the attributes of each of the choices made. All covariates met the normality assumption following log-transformation. The analysis on 3019 observations was carried out in the SAS package PROC MDC [35] using quasi-Newton optimisation and 100 Halton draws.

### The definition of choice set

**Figure 3:**
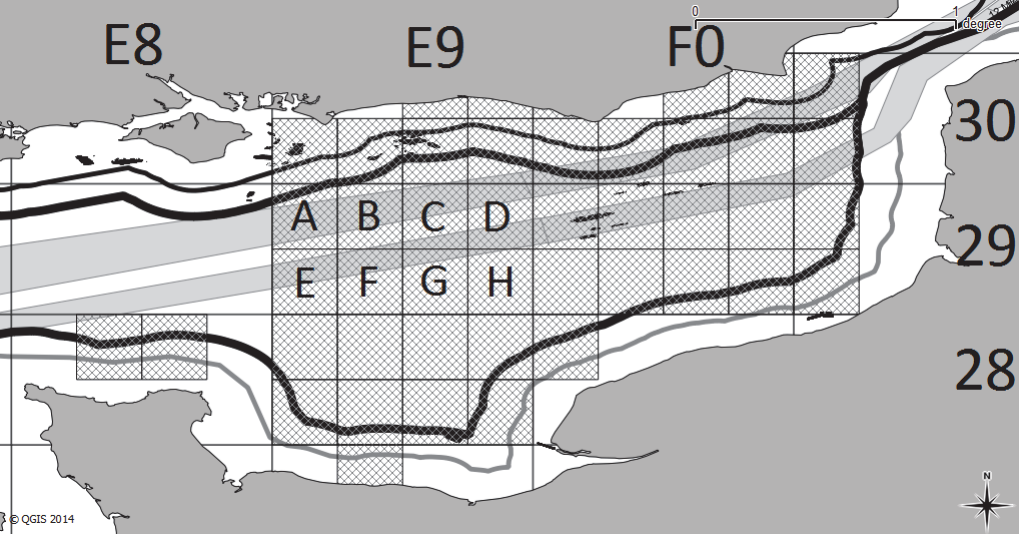
The eastern English Channel with ICES rectangles overlaid and the choice set represented by the hatching geo-referenced by ICES rectangle and the eight sub-rectangles within.

When designing RUMs, fisheries scientists are confronted with the problem of creating a choice set, which covers the individual sites to which a fisher travels to fish. If sites are too small (individual latitude/longitude positions), there may not be sufficient site-specific information, but if they are too large, important site-specific information can be lost when aggregating, losing information valuable to policy-makers. Fishers have prior knowledge of resource distribution and habitat [36], [37], and scallops are relatively static molluscs, suggesting that in future years, any choice set will be subject to relatively little change. On the basis of this assumption, the predetermined area making up the choice set for this study was based on the 2005–2010 effort distribution of scallop dredgers plotted from the VMS records (Fig. 2). A trade-off in scale was necessary so the dataset was grouped into 45 sub-rectangles, determining the choice set (Fig. 3). Having too many specific choices can reduce the stability of standard algorithms for maximum likelihood estimation as the number of alternatives rises past around 50 due to data multicolinearity [38].

### Variable selection

Net benefit or profit per set of fishing trips is not easily computed as detailed cost data (variable and fixed) is costly to collect and such confidential information is not usually disclosed. Researchers therefore use a proxy of value per unit effort (vpue) rather than cost, which relates to the net benefit of variations in stock density [39], [11]. Value per choice was calculated as a proportion of the total value (revenues from landings) per ICES rectangle based on effort (hours fished) derived from the VMS, and vpue was then computable. The average vpue by year, month and location was calculated for the fleet and lagged both by month and annually to take account of spatial fluctuations at different temporal scales. The past percentage of a particular vessel’s scallop trips to a fishing location as a percentage of the fleet total elsewhere was used to represent fisher habit/experience and to track the seasonal nature of the fishery, as in [40], and was also lagged as above.

Where possible, fishers are assumed to maximise their returns [41]. Subject to the weather and other factors, they trade off travel costs against the quality of the fishing grounds. A proxy for perceived costs was calculated based on the average fleet distance to landing port from VMS fishing locations, calculated using the Haversine formula [42], weighted by mean average fuel price from fishing in the same location in the same month of the previous year fishing (i.e. lagged average costs). [43] survey of fishers in the south west of England showed that fishers routinely keep track of fuel prices in order to forecast their potential earnings after deductions for other costs. Landing port was used as it was assumed that the fishers would have prior knowledge of seasonal market prices in the proximity of fishing locations.

Aggregate mining activity enters the model as the average percentage coverage of this mining activity in the location the previous month (to capture potential past activity as a nuisance to fishing operations). The 6 mile limit (as a proxy for the English restricted zone for certain vessels over 14m) and the 12-mile limit (as a proxy for the French internationally restricted zone) were treated as a spatial constraint. Maritime traffic was included as average hours in which a location was occupied by marine shipping traffic in the previous month. Finally, as a proxy for congestion and social influencing effects, we included the average hours fished the previous month by English, French and other (unidentified fishers grouped) fishing vessels. The variable selection set was merged with individual scallop trip data by year, month and location, such that for every trip, the decision-maker had a choice of the specified 45-subrectangles (Table 1). Based on the historic time-series of VMS data, fishing activity was ascribed to a particular sub-rectangle, and values took a value of 1 if a location was selected or 0 otherwise It is important to note that for any particular vessel and any given trip, a number of observations may exist in a number of different sub-rectangles, hence each choice is considered separately as components within a fishing trip).

**Table 1:** Definition of variables used in the RUM to model fisher location choice for the 45 ICES sub-rectangles in the eastern English Channel as defined in Fig. 3.

**Variable**	**Description**
Effort (yr)	Percentage of trips to the location in the same month as the previous year.
Effort (m)	Percentage of trips to the location in the previous month in the current year.
VPUE (yr)	Average vpue of scallops from fishing in the same location in the same month in the previous year, in £’s per hours fished.
VPUE (m)	Average vpue of scallops from fishing in the same location the previous month in the current year, in £’s per hours fished.
Traffic	Average hours occupied by marine traffic the previous month in the current year.
Aggregate	Average % coverage of area occupied by aggregate activity the previous month in current year.
Fleet (English, French, Other)	Average hours occupied by fishing activity by English, French and other fleets the previous month in the year of fishing.
Cost	Average distance to landing port multiplied by the fuel price the previous year (£).
12mile	Average % coverage of the location by French 12 mile limit.
6mile	Average % coverage of the location by English 6 mile limit.

### Sensitivity analysis

To test the sensitivity to different variables the mean choice probabilities were calculated from the model output and then compared with mean choice probabilities after re-running the model under alternative scenarios where each variable was doubled/halved one at a time. The differences in probability of location choice, under each of these scenarios, show the magnitude of the effect on location choice and how sensitive the variables are to changes i.e. how the variables that penalise fishing operations (e.g. aggregate extraction, marine traffic, and fuel costs) affect fishers, in contrast to expected vpue which should encourage fishing operations.

## Results

The mixed model showed a McFadden’s pseudo-*R* ^2^ of 0.19, suggesting a very good fit [44]. Theoretically, the range for McFadden’s pseudo-*R* ^2^ is between 0 and 1, but the general rule of thumb is that any value from 0.2 to 0.4 suggests an excellent fit, comparable to an ordinary least squares (OLS) *R* ^2^ of 0.7–0.9 [45].

All mixed model coefficients were statistically significant (*p* < 0.01) except the coefficient for the average vpue of scallop from fishing in the same location in the same month as in the previous year and the proxy for congestion/social influence in the previous month of the current year for the French fleet (Table 2). The estimated standard deviations of the estimates were not significantly different from the mean (indicating that the parameters do not vary significantly in the population of fishers) for past vpue, cost, average percentage coverage by marine traffic and average hours occupied by fishing activity by English/other fishing vessels. Conversely, the effort to the location in the previous month in the current year, the average percentage coverage by aggregate activity and the average effort in the same month the previous year did vary, perhaps relating to variations in characteristics of the fishers not captured in the model.

**Table 2:** Estimated parameter values, where the dependent variable took a value of 1 if a choice was made or 0 otherwise.

**Parameter**	**Estimate**	**SE**	**Significance**
traffic_m	−0.1589	0.0299	***
traffic_s	0.0257	0.3244	
VPUE (yr)_m	0.0109	0.0231	
VPUE (yr)_s	0.1677	0.0715	**
VPUE (m)_m	0.1317	0.0218	***
VPUE (m)_s	0.0708	0.1289	
Effort (yr)_m	0.1479	0.0429	***
Effort (yr)_s	0.8723	0.0985	***
cost_m	−0.2184	0.0421	***
cost_s	−0.0105	0.4042	
Effort (m)_m	0.9894	0.0432	***
Effort (m)_s	0.8238	0.0786	***
aggregate_m	−0.0955	0.0156	***
aggregate_s	−0.3503	0.0513	***
6 mile †	−0.3133	0.0863	***
12 mile †	−0.2206	0.1097	**
fleet (English)_m	0.0364	0.006452	***
fleet (English)_s	−0.005047	0.0443	
fleet (French)_m	−0.004345	0.009317	
fleet (French)_s	−0.0412	0.0406	
fleet (Other)_m	0.0214	0.006978	***
fleet (Other)_s	−0.009928	0.0562	

Parameters marked _m are the normal mean coefficients and _s are between-population standard deviations. Note: The coefficients for variables marked † are assumed to be fixed to allow for the fact that the probability of visiting a larger less restricted choice is higher than that for a smaller more restricted choice, all else equal, hence having this variable vary over fishers would not be meaningful [55]. df = 1 in all cases. Statistical significance at * 10% level, ** 5% level, and *** 1% level.

The effort distribution maps in Fig. 2, coupled with the model results (Table 2) show how the scallop dredges interact with the shipping traffic separation scheme, aggregates and fisheries outside the English 6 and French 12-mile limits. In general the mean coefficients show the signs one would expect: English scallop fishers are negatively affected by the English 6 and French 12 mile restrictions, as well as aggregate activity and marine traffic. Despite this, in every year of the study there was a large amount of fishing effort in these areas, even more so in 2010 within the high-traffic area, perhaps because of a trade-off with larger expected vpue in these areas. There is a significant positive influence of vpue in the previous month on the tactics of fishers, but not of vpue in the same month of the previous year, which strongly suggests in-year variability is the key driver of behaviour. In contrast, cost was a negative influence as expected. Past effort variables, which were included to depict habit or knowledge of past success of fishing grounds, have positive coefficients, suggesting they are important drivers of fisher location choice.

As part of a ‘what if analysis’ a series of numerical simulations revealed that fishers responded to a 50% decrease in % area covered by aggregate extraction differently depending on the spatial position of the activity. In an area close to shore associated with aggregate extraction (30E9G), there was a relatively large difference in probability of fishing, of +0.012, while there were small noticeable increases in nearby areas 30E9F and 29F0C. Doubling the coverage of aggregate extraction resulted in fishers moving out of these locations (notably these same areas, 30E9G, 30E9F and 29F0C) with a change of probability of −0.018, −0.012 and −0.004 respectively. Fishers moved into a more offshore site of existing extraction (29F0B), which recently has contained very high fishing effort, with a change in probability of +0.014. These observations suggest that aggregate mining activity heavily influences fisher decision making, possibly due to knowledge of the habitat that scallops live in, coupled with past experience (Fig. 2).

**Figure 4:**
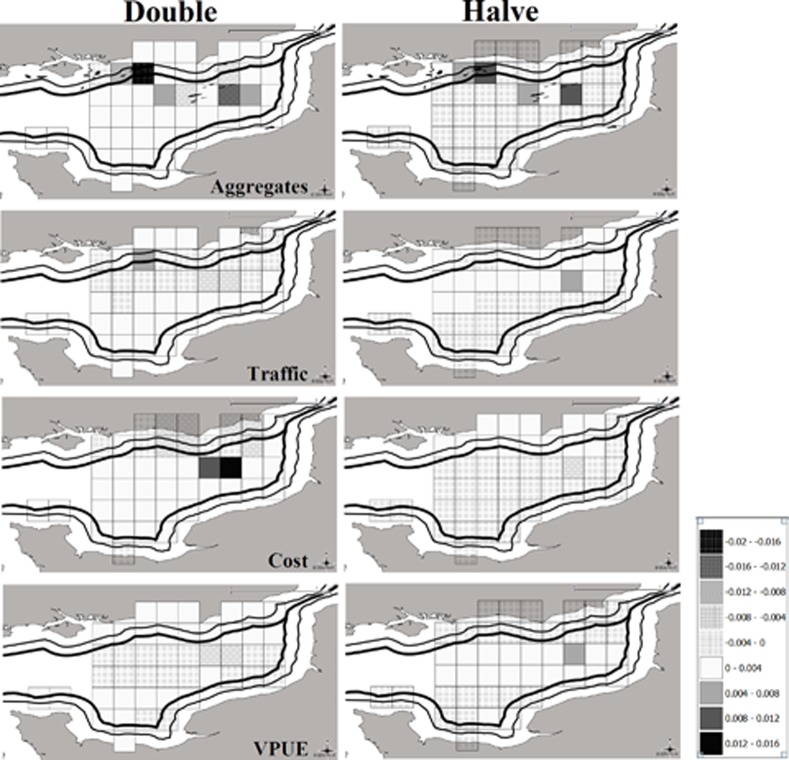
Changes in probabilities when halving or doubling each variable in contrast to the benchmark model’s observed variable mean values.

Most of the main scallop grounds are located within busy marine traffic areas (Fig. 2) and therefore one would expect that with a decrease in traffic intensity there would be less competition for space and fishers would move into these areas. Maritime traffic, however, showed relatively small effects. Doubling the coefficient of maritime traffic intensity resulted in fishing effort being displaced out of the traffic lanes, essentially spreading out, whereas halving the coefficient led to an increase in predicted effort into the traffic lanes, most notably 29F0A, 29F0B and 29F0C. Changes in expected fuel cost did not result in large significant differences in probabilities of site choice. When fuel prices are halved fishers move closer in shore to the English ports, in contrast when they are doubled fishers move to areas offshore where the concentration of fishers and expected vpue is at its highest (e.g. areas, 29F0B, 29F0C and 29F0D) or nearer to French landing ports, resulting in a ‘complimentary effect’ with expected costs and expected vpue (Fig. 4).

## Discussion

It is widely recognised that decision-makers and managers desire an ecosystem-based approach to address interlinked drivers of social well-being [46]. Marine Spatial Planning necessitating the balancing of multiple objectives; fisheries managers need to understand the implications of effort displacement from closing an area and the unforeseen consequences of their management actions (e.g. effects on other marine life, economic implications and effects on other maritime sectors). Several authors have stressed the importance of anticipating fisher behaviour in response to management regulation, in order to reduce implementation error [47]–[49]. Here, a mixed logit RUM was applied at fine-scale resolution to assess the key determinants of scallop fisher behaviour in the eastern English Channel, so that if a regulation or new activity emerges, fishing effort re-allocation can potentially be predicted.

A key finding was that past fishing success in a location within the previous month was a good predictor of continued fishing in that location. This can be interpreted as a proxy for habit, knowledge or experience, as in other studies [40], [10]. Similarly, the expected marginal net revenue of visiting one fishing site rather than another, in terms of vpue, was significant as expected [50]. This is more apparent for the vpue in the previous month, rather than in the same month the year before, potentially capturing either seasonality or more likely short-term temporal correlations in stock abundance (see Table 2). Surprisingly, perceived fuel costs were not a major driver in choice of fishing grounds, possibly because of the proximity of grounds to landing ports in the eastern English Channel. The French12-mile limit and English 6 mile limit unsurprisingly had negative influences on fisher site choice, possibly because of productive fishing grounds within limits, which are rendered unavailable to the scallop vessels. Nevertheless competition from the inshore national fleet could become an issue if the fleet is forced to occupy a reduced spatial geographic footprint than was previously the case, for example by spatial closures. Of further policy importance are the effects of other commercial maritime activities (e.g. transport, aggregates mining) on the behaviour of the scallop fleet. If interactions with these sectors are better characterised then the implications for the scallop fleet of other maritime sectors can be assessed in advance.

The analysis indicates that this fleet exhibits some risky behaviour in their responses, as the mean of the coefficients determining site choice and the estimated standard deviation of the coefficients in Table 2 show highly significant estimates of some of the drivers, suggesting that the parameters vary within the wider population of fishers [40]. The signs of the standard deviations in some instances are negative, but for estimation purposes they are free to take any sign, because the normal distribution is symmetrical around its mean, and the absolute value can be taken to estimate the variance. For the coefficients that do vary between fishers (i.e. previous effort (last month and last year) and presence of aggregate extraction) we can assess what proportion of the population of fishers see these factors as positive or negative when making decisions about site choice. Taking the mean and standard deviation together the point estimates of the coefficients can be calculated by standardising the scores (z scores) and thus a probability can be calculated. The model suggests that 88% of the population of fishers see effort in the past month as a positive inducement to fishing in the same location again and 12% see it as a negative inducement, which will be dependent on the time they spent in the location previously and their success in terms removing the harvestable biomass, if they ‘fished out’ an area they are unlikely to return. Similarly past effort in the same month of the previous year is a positive influence on location choice for 58% of fishers. The areas occupied by aggregates mining are chosen more than expected with about 40% preferring fishing in these areas, in contrast to the other 60% seeing it as a negative influence, confirming the assumption that the aggregate industry does impact scallop fishing. [15] found that by setting aside marine areas for aggregates mining, this resulted in reduced fishing effort. Since 2005, aggregate extraction licences have been granted over large areas [51]. This is contrary to [52] findings for sole, which suggests that aggregate mining can have a positive effect on the catchability of sole by beam trawlers and hence on profitability. Perhaps, increased turbidity increases sole catchability (by reducing visual cues for escape and/or fish being disturbed from the seabed) or the dispersal of food into the water column encourages sole to move away from the bottom to feed or they may favour the previously mined area because of changed food resources or substrate. In stark contrast however, a recent study by [53] using time series cross correlation approach concluded that aggregate extraction activity, proximity and intensity didn’t have any impact on fisher activity. The differences could be attributed to the different statistical approaches employed. In the approach adopted by [53], only one sector of activity was investigated, in contrast to this study whereby two competing sectors were studied with the inclusion of economic data. ARIMA models (such as those employed by [53]) do not take account of individual behavioural interactions and are purely based on past time series behaviour, however they remain an excellent tool to support expert judgment.

The shipping Traffic Separation Scheme (TSS) in the English Channel controls one of the busiest shipping lanes in the world and attempts to mitigate against the possibility of maritime accidents, but can also impede fishing. The output from the model suggests that the presence of a TSS significantly reduces the probability of a fisher choosing a location, suggesting that the policy is having the desired effect of separating fishing from other activities, though at the cost of reduced ability to choose areas of potential high profitability.

This study gives clues to policy makers about the likely impact of their actions on fisher behaviour. For example, an increase in traffic densities would have a high chance of displacing effort to local inshore waters (Fig. 4). Conversely, the fleet responds to higher fuel cost by going further offshore with the expectation of the reward of higher returns, and when costs are lower they fish equally between inshore and offshore locations as they are not forced to cover higher costs by fishing in areas with highest vpues. Fig. 4 suggests that when aggregate mining is doubled there is a greater increase in fishing activity offshore and when halved there appears to be movement of fishing into the location of extraction. Also of note is the movement of vessels towards the French 12 mile limit, resulting in shorter distances to land to French ports.

A further important observation is that if one of the parameters that disadvantages fishers’ (e.g. shipping traffic densities) is altered, the competition for space effectively increases and the fishery spreads out. This may be because the traffic lanes are home to the best scallop fishing grounds and the specific location a vessel relocates to is the one with the next best trade-off between expected catch rates and distance to landing ports. This is also apparent for the competition with the aggregate sites, which are located in the heart of good scallop fishing grounds. Any reduction of the space taken up by aggregate mining, especially inshore results in an increase in effort allocation to those locations. This “fishing for space” where observed, could be viewed as symptomatic of competition within the fleet as well as a response to other sectors, and hence this could be used as a direct measure of spatial conflicts.

## Conclusions and Future Work

The Eastern English Channel is a shared resource and there is increasing competition for space and resources, requiring novel management approaches that account for all or some of the interactions between sectors. To our knowledge, no other study has used a mixed RUM at fine resolution to assess key determinants of human behaviour in relation to different maritime sectors and as a possible tool for MSP. The results are promising and lay the foundations for future work that could include adding Marine Conservation Zones (MCZs) to the model. Final decisions on where MCZs will be introduced in the English Channel and what activities will be excluded are still to be clarified or have only recently been resolved, so it was not appropriate to incorporate simulated closures into the model. Nevertheless, the approach taken could be applied to other fleets, as RUMs offer the capacity to model individual behaviour at fine spatial and temporal scales needed for assessing the implications of policy decisions [54]. It would also be desirable to re-fit the data to recent data where fishing effort is more stable (during the investigated time period effort gradually increased) and as such results could appear somewhat different. Further work might include the Scottish fleet which represent a large proportion of scallop fishing effort in the eastern English Channel, evaluating trade-offs with both socio-economic and conservation objectives using efficient and effective spatial planning tools such as Marxan and MinPatch.
