# Correction: Fishing for Space: Fine-Scale Multi-Sector Maritime Activities Influence Fisher Location Choice

**DOI:** 10.1371/journal.pone.0121498

**Published:** 2015-03-19

**Authors:** The PLOS ONE Staff

There is an error in the first sentence of the “The UK scallop fleet” subsection of the Materials and Methods. The correct sentence is: The UK scallop (Pecten maximus) industry is one of the UKs most valuable fisheries and was valued at >£66.9 million (58000 tonnes), £16 million in the Channel alone in 2012 [18], employing 600 people in the UK catching sector out of a total of 13000, and 750 people out of a total of 17 000 in the processing sector [19].
